# Assembly and Functional Analysis of an S/MAR Based Episome with the Cystic Fibrosis Transmembrane Conductance Regulator Gene

**DOI:** 10.3390/ijms19041220

**Published:** 2018-04-17

**Authors:** Davide De Rocco, Barbara Pompili, Stefano Castellani, Elena Morini, Luca Cavinato, Giuseppe Cimino, Maria A Mariggiò, Simone Guarnieri, Massimo Conese, Paola Del Porto, Fiorentina Ascenzioni

**Affiliations:** 1 Department of Biology and Biotechnology “Charles Darwin”, Sapienza University of Rome, 00185 Rome, Italy; davide.derocco@uniroma1.it (D.D.R.); barbara.pompili@uniroma1.it (B.P.); morinielena.em@gmail.com (E.M.); luca.cavinato@uniroma1.it (L.C.); 2 Laboratory of Experimental and Regenerative Medicine, Department of Medical and Surgical Sciences, University of Foggia, 71122 Foggia, Italy; stefano.castellani@unifg.it (S.C.); massimo.conese@unifg.it (M.C.); 3 Department of Pediatrics and Infant Neuropsychiatry, Centro di Riferimento Fibrosi Cistica Regione Lazio, Sapienza University, 00165 Rome, Italy; giuseppe.cimino@uniroma1.it; 4 Laboratory of Functional Biotechnology, Center of Sciences on Aging and Translational Medicine (CeSI-MeT), Department of Neuroscience, Imaging and Clinical Sciences, University G. d’Annunzio of Chieti-Pescara, 66013 Chieti, Italy; mariggio@unich.it (M.A.M.); guarnie@unich.it (S.G.)

**Keywords:** episome, S/MAR, cystic fibrosis, CFTR, gene therapy

## Abstract

Improving the efficacy of gene therapy vectors is still an important goal toward the development of safe and efficient gene therapy treatments. S/MAR (scaffold/matrix attached region)-based vectors are maintained extra-chromosomally in numerous cell types, which is similar to viral-based vectors. Additionally, when established as an episome, they show a very high mitotic stability. In the present study we tested the idea that addition of an S/MAR element to a CFTR (cystic fibrosis transmembrane conductance regulator) expression vector, may allow the establishment of a CFTR episome in bronchial epithelial cells. Starting from the observation that the S/MAR vector pEPI-EGFP (enhanced green fluorescence protein) is maintained as an episome in human bronchial epithelial cells, we assembled the CFTR vector pBQ-S/MAR. This vector, transfected in bronchial epithelial cells with mutated *CFTR*, supported long term wt *CFTR* expression and activity, which in turn positively impacted on the assembly of tight junctions in polarized epithelial cells. Additionally, the recovery of intact pBQ-S/MAR, but not the parental vector lacking the S/MAR element, from transfected cells after extensive proliferation, strongly suggested that pBQ-S/MAR was established as an episome. These results add a new element, the S/MAR, that can be considered to improve the persistence and safety of gene therapy vectors for cystic fibrosis pulmonary disease.

## 1. Introduction

Since the identification of cystic fibrosis (CF) disease in 1938 and the discovery of the causative *CFTR* (*CF transmembrane conductance regulator*) gene in 1989, life expectancy of CF patients has increased progressively to more than 40 years in developed country. This improvement can be assigned to the development of interventions aimed to augment airway clearance, nutrition, and to reduce microbial infections [1]. However, despite the advancements in understanding how mutated *CFTR* causes abnormal ions transport, therapies that correct the basic CF defect are very limited. High-throughput screening approaches led to the identification of two small molecules that restore CFTR-mediated ion transport in CF cells. The first was a potentiator, VX-770 (ivacaftor), that restores CFTR activity in class III mutations by improving channel opening, in particular in patients with Gly551Asp mutation. Clinical trials showed that, compared with placebo, ivacaftor improved lung function, as assessed by FEV_1_ (forced expiratory volume in 1 s) by about 10%, reduced sweat chloride concentration and pulmonary exacerbations [1,2]. The second small molecule, which was specifically identified as a corrector of Phe508del CFTR was VX809 (lumacaftor); this drug improves processing and intracellular trafficking of the Phe508del CFTR to the cell surface leading to enhanced chloride transport in vitro [2]. However, in clinical trials lumacaftor alone failed to show any significant benefit in CF patients homozygous for Phe508del mutation [3,4]. More recently, the combinatory therapy ivacaftor/lumacaftor was shown to induce a modest improvement of lung function in homozygous Phe508del patients [5]. Possibly, this may be due to the destabilizing effect of VX-770 on CFTR, which has been observed either in non-CF and CF epithelial cells [6,7]. Overall, it appears that the development of efficient CFTR-repairing molecules is still a big task with very limited success, so far.

CF is a single gene disorder caused by mutations in the *CFTR* gene, supporting the notion that the introduction of the wt copy of the gene would prevent CF disease. This approach has the advantage to be mutation-independent and applicable to all CF patients. Additionally, as mutations in the *CFTR* gene have been causatively linked to CF [8], development of genetic medicine is possible even though the disease pathophysiology is not completely understood. This is particularly relevant as it is still debated how CFTR dysfunction causes CF. In addition to the well-established “low-volume hypothesis” predicting that CF lung disease is mainly due to low Cl^-^ efflux and Na^+^ hyper-absorption in airway epithelial cells [1], it has been claimed that loss of bicarbonate secretion and altered pH impair mucus function and innate defense mechanisms [9,10]. Independently from the basic mechanism of altered ion fluxes, the CF airway epithelium displays other defects, including actin and tight junction disorganization linked to the alteration of the NHERF (Na^+^/H^+^ exchanger regulatory factor)-1 multiprotein complex which tethers CFTR on the apical membrane [11,12,13].

Genetic medicine may comprise gene therapy (GT), gene/cell therapy and genome editing. To date, 27 Phase I/II CF gene therapy clinical trials, involving 600 patients, have been performed worldwide [14,15]. Clinical efficacy in three of these studies (155 participants in total; comparison between gene therapy to placebo; different designs and gene transfer agents) has been reviewed leading to the conclusion that, at the moment, there is no gene transfer protocol for the treatment of the CF lung disease [16]. More recently, completion of a double-blinded placebo controlled multi-dose trial conducted on 116 patients with a non-viral vector, showed a significant, although modest, improvement of lung function, as determined by FEV_1_ (forced expiratory volume in 1 s measurements but no significant increase of ion transport and any detection of vector specific CFTR mRNA [17]. Different hypotheses have been postulated to explain this discrepancy including timing and sensitivity of the assays and possibly local differences in the vast area of the lung [15].

GT approaches in CF have been conducted with a wt copy of the *CFTR* gene controlled by an exogenous promoter and delivered to the lung by a replication-defective viral vector, with tropism for airway cells, or by synthetic vectors. Among the viral vectors, adenovirus (Ad), which appeared promising in preclinical studies, revealed important limitations due to acquired and pre-existing immune responses [18]. Other viral vectors under investigation include adeno-associated virus (AAV) and lentivirus, both of which are expected to mediate integration of the therapeutic gene [19,20,21,22]. However, while integration of AAV is site-specific and considered safe [21], lentiviruses shows preferential integration into transcription units. According to the prevalent tethering model, this is achieved by the activity of the cellular factor LEDGF (lens epithelium-derived growth factor)/p75, which recruits the lentiviral integrase to active transcription units [23,24]. This opens the possibility to re-direct lentiviral integration by creating artificial LEDGF/p75-based proteins with sequence specific DNA binding domains, such as the phage lambda repressor, or by modulating the expression of the tethering factor LEDGF/p75 reviewed in [24]. Indeed, it has been shown that in LEDGF/p75-depleted cells, lentiviral integration occurs more frequently near transcription units and CpG islands [23]. Nonetheless, the genotoxicity risk associated to vector integration is expected to be low in airways epithelia compared to proliferating tissues, as they are terminally differentiated [25,26]. A very attractive tool to precisely correct gene mutations is undoubtedly the CRISP/Cas9 mediated genome editing, which has been recently shown to correct *Fah* mutation in vitro and in an in vivo murine model [27].

Non-viral vectors consist of DNA, in many cases plasmid DNA containing the gene of interest, which are usually delivered in complexes with cationic lipids or polymers. The complexes interact with the cellular plasma membrane and mediate DNA entry and trafficking to the nucleus. Non-viral vectors have the advantage to minimize the risk of immunogenicity thus allowing repeated administration. However, as plasmids do not possess sequences allowing DNA replication and maintenance in mammalian cells, their persistence is limited in time and at the best, may only result in transient complementation of the *CFTR* gene mutation. Accordingly, the *CFTR* gene-liposome complexes (pGM169 plasmid and the gene transfer agent GL67A) were administered monthly for the entire 12 months duration of the trial [15]. Whether and how, plasmid instability, impacts on the efficacy of non-viral gene medicine has not been properly investigated yet, as it would require the use of plasmid vectors equipped with non-viral stabilizing and/or replicating elements.

S/MAR (scaffold/matrix attached region) elements were identified in sequence analysis of various mapped mammalian origins of replication and subsequently shown to support episomal replication of plasmids in mammalian cells [28]. The first S/MAR-based vector shown to persist in either immortalized or primary cells, was the pEPI [29]. When examined, S/MAR vectors have been been found to stabilize as episomes in only few percentage of the transfected cells, by an unknown mechanism, possibly involving interaction of the vector with specific chromosome territories [28,30]. This limitation is counterbalanced by the fact that, in established cellular clones, the pEPI vector localizes exclusively in interchromatin space and associates with early replication and actively transcribed foci [30,31]. This may contribute to the long-term persistence of pEPI-derivatives that was been reported in vitro and in vivo in animal models including large animals as the pig [32]. In different murine models, S/MAR episome persistence was demonstrated in the liver and in retinal pigment epithelial cells [33,34,35]. Interestingly S/MAR elements have been also integrated into viral vectors giving rise to hybrid systems that, following cell infection, are converted into autonomous episomes [36].

Therefore, S/MAR episomal vectors by supporting long-term gene expression and episomal persistence are expected to counteract some important limitations of non-viral GT approaches such as transient gene expression and vector loss while being free of immunogenicity and insertional mutagenesis.

Here we present data obtained with an S/MAR-stabilized plasmid encoding the *CFTR* gene. The functional analyses of the S/MAR-CFTR vector showed improved plasmid stability, which positively impacts CFTR expression in bronchial epithelial cells and assembly of tight junctions as assessed by measurement of trans epithelial resistance and immunocytochemistry.

## 2. Results

### 2.1. Long-Term Maintenance of pEPI-EGFP in Epithelial Cells

**Figure 1:**
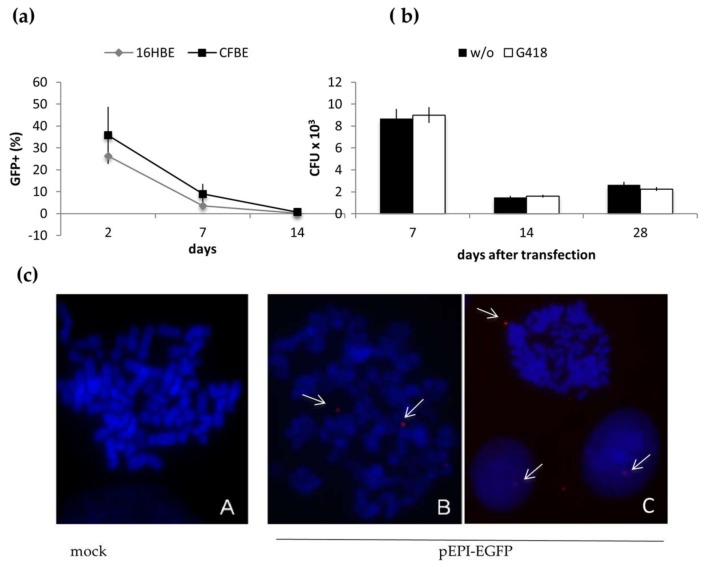
Long-term maintenance of the pEPI-EGFP episome in bronchial epithelial cells. (**a**) percentage of cells expressing GFP (GFP^+^) in 16HBE and CFBE populations transfected with the pEPI-EGFP by nucleofection, and propagated in non-selective conditions. Samples were analyzed by flow cytometry at the indicated times after transfection (mean ± SD, *n* = 2). (**b**) *E. coli* rescue from transfected CFBE cells. Hirt extracts were prepared from transfected cells propagated for 7, 14 and 28 days, with (G418) or without (w/o) selection; the pEPI-EGFP was then recovered in *E. coli*. Data (mean ± SD) refer to the total number of CFU recovered in DH10B electrocompetent cells using 2 μL extracts; control electroporation yielded 5 × 10^3^ ± 50 CFU/10 pg plasmid DNA (pMAX). (**c**) Metaphase chromosomes hybridized to the pEPI-EGFP probe (red). **A**, un-transfected cells (mock); **B** and **C** representative images of cell transfected with pEPI-EGFP and analyzed 14 days after transfection; episomes are highlighted by white arrows; dots outside the nuclei were not count; images were taken at 630× magnification.

First, we evaluated whether the S/MAR element could promote episomal replication and mitotic stability of plasmid-based vectors in human bronchial epithelial cells. For this, the 16HBE14o^−^ (16HBE) and CFBE41o^−^ (CFBE) cell lines were transfected with the S/MAR-based vector pEPI-EGFP and maintained in culture for one month. Two days after transfection GFP-positive cells in the bulk population were about 25% and 35% in 16HBE and CFBE, respectively (Figure 1a). In both cell lines this population progressively declined to less than 10% after one week and below 1% after two weeks (Figure 1a). This result could be due to loss of the vector or, as previously reported [28], to silencing of the GFP expression. We tested the former hypothesis by tracking the vector in dividing cells for about one month after transfection using the *E. coli* rescue assay. Additionally, cells expanded without or with plasmid-encoded selection (resistance to G418) were analysed. The CFU (colony forming unit), which represent the amount of episomal vector within the cells, were quite high at day 7, then decreased (about four-fold) at day 14 and remained stable till day 28. No difference was observed between cells grown with or without selection (Figure 1b). Next, to further analyze the episomal maintenance of the pEPI-EGFP, transfected cells were analyzed by fluorescence in situ hybridization (FISH). This analysis was limited to cells propagated in non-selective conditions up to 28 days after transfection. Representative images are reported in Figure 1c, which clearly shows the presence of discrete episomes within the metaphases. In most cases one, rarely two, episomes per cell were detected whereas, no integration events were observed. In the attempt to determine the cells containing the pEPI-EGFP and those without, we observed that in many cases hybridization signals resembling the vector, could not be assigned to specific metaphases. Therefore, to circumvent this problem, we analysed interface nuclei, which clearly showed the presence of distinct hybridization signals within the nuclei as reported in Figure 1c (panel C). Preliminary analysis validated this approach as a similar number of cells containing the pEPI-EGFP was detected in metaphases and nuclei Thus, taking into account at least 50 nuclei for each condition, the episome was found in 80% and 40% of the cells at day 7 and 14, respectively.

Globally, these results suggest that the pEPI-EGFP is maintained as an episome in growing bronchial epithelial cells, not only under selection, but also in non-selective conditions. Importantly, we did not observe vector integration events.

### 2.2. Assembly and Analysis of the S/MAR-Based CFTR Vector

Based on the results obtained with the pEPI-EGFP in growing bronchial epithelial cells, we assembled a CFTR episome by cloning the S/MAR element into the CFTR vector pBQ6.2 [37]. The resulting vector pBQS/MAR (Figure S1) was analysed in CFBE cells, homozygous for the Phe508del *CFTR* allele [38].

First, we studied the episomal maintenance of the vector in transfected cells propagated without selection up to two weeks. We selected this experimental condition based on the following consideration: (i) similar amounts of pEPI-EGFP were recovered from transfected cells 14 and 28 days after transfection; (ii) the episomal maintenance of the pEPI-EGFP was not very much different between the selective and non-selective growth; (iii) in vivo gene therapy protocols, such as those used for the CF lung gene therapy, do not apply selective strategies for vector maintenance. CFBE cells were transfected with pBQ-S/MAR, or the parental plasmids pBQ6.2 and pEPI-EGFP by lipofection and the efficiency of transfection was determined by flow cytometry 48 h after transfection. The percentage of GFP + cells was 14.58 ± 0.72 (mean ± SD; *n* = 3) for CFBE/pBQ-S/MAR and 31.43 ± 7.48 (mean ± SD; *n* = 3) for CFBE/pEPI-EGFP. Comparison of transfection experiments performed with equimolar amounts of the different vectors, or with the same amount of vector DNAs (in the range 3–10 μg/10^6^ cells), failed to significantly reduce differences in transfection efficiency between pBQ-S/MAR and pEPI-EGFP suggesting that large vectors, such as pBQ-S/MAR (12.4 kbp), transfect CFBE41o- cells less efficiently.

**Figure 2:**
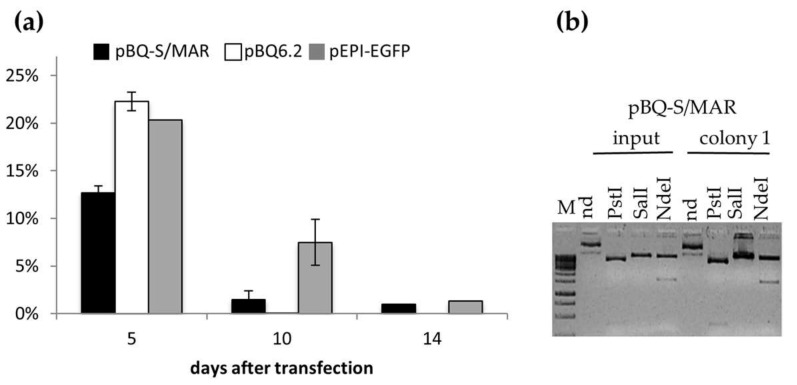
The pBQ-S/MAR vector is maintained as an episome in dividing epithelial cells. (**a**) Percentage variation (mean ± SD, *n* = 3) of *E. coli* transformants at days 5, 10 and 14 with respect to day 2. (**b**) Restriction profile of pBQ-S/MAR (input) and plasmid recovered from CFBE cells at day 14 (colony 1). M, 1 kb molecular weight marker; nd, not digested.

Next, cells were grown up to 14 days, corresponding to seven cell duplications and, at time intervals, low molecular weight DNA was extracted by the Hirt method. Hirt extracts were used to transform *E. coli* by electroporation. The colony forming units (CFU) obtained with 2-day-Hirt extracts were used to normalize those obtained with the 5-, 10- and 14-day samples (Figure 2a). Similar to the pEPI-EGFP in 16HBE14o- cells, pEPI-EGFP and pBQ-S/MAR were progressively lost during cells growth, but still present 10 and 14 days after transfection. On the contrary, the parental pBQ-6.2, was undetectable from day 10 onwards. At the end of the experiment, day 14, irrespective of the initial difference between pEPI-EGFP and pBQ-S/MAR, the number of bacterial clones recovered from pBQ-S/MAR- and pEPI-EGFP-Hirt extracts attained at similar level, suggesting that a similar fraction of cells retained the vector.

To check the integrity of the vectors recovered from transfected cells, plasmids were extracted from at least 3 bacterial colonies at each time point analyzed, and analyzed by restriction digestions. This analysis showed that the recovered plasmids possessed the same restriction profile as the input ones. An example of this analysis is shown in Figure 2b reporting *Pst*I, *Nde*I and *Sal*I restrictions of pBQ-S/MAR recovered at day 14, as compared to the input pBQ-S/MAR vector. These results suggest that the plasmid structure is stably maintained in epithelial cells, although, the presence of subtle sequence variations cannot be ruled out.

### 2.3. CFTR Expression and Function in CFBE Cells

**Figure 3:**
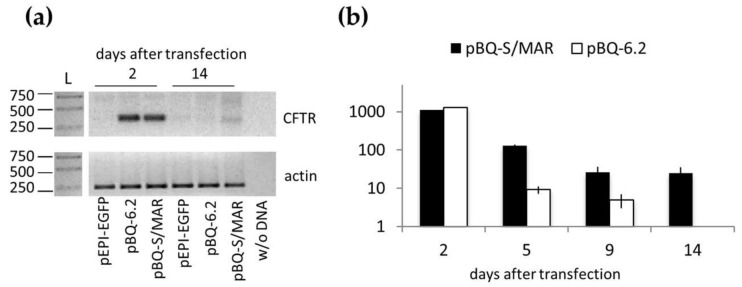
CFTR expression in CFBE cells. (**a**) Reverse transcription-PCR for wt CFTR (upper panel) and β-actin (lower panel) of CFBE cells transfected with the indicated vector propagated for 2 and 14 days after transfection. Samples were separated by agarose gel electrophoresis in 1.5% agarose gels; L, ladder (band size is in bp). (**b**) CFTR mRNA relative quantification (RQ) in the indicated samples. RQ (mean ± SD, *n* = 2, each in duplicate) was determined by the ΔΔ*C*_t_ method using parental CFBE cells as calibrator.

The level of CFTR transcripts was examined in transfected cells at day 2 and 14 after transfection. This was done in cells transfected with pBQ-S/MAR and the control plasmids pBQ-6.2 and pEPI-EGFP. As reported in Figure 3a, CFTR mRNA was abundant in CFBE-pBQ-S/MAR and -pBQ-6.2 at day 2 then, at day 14, the transcript decreased to almost undetectable level in all samples but remained clearly detectable in pBQ-S/MAR-containing cells. This result was further supported by a time-course evaluation of *CFTR* expression by real time PCR (Figure 3b). CFTR expression in CFBE-pBQ-6.2 progressively declined to undetectable level, while that CBFE-pBQ-S/MAR was stabilized from the ninth day onward. Notably, similar levels of CFTR transcripts were observed at the beginning of the experiment (i.e., two days after transfection) in CFBE cells transfected with pBQ-S/MAR or pBQ-6.2, strongly supporting the notion that long term CFTR expression is due to vector persistence.

**Figure 4:**
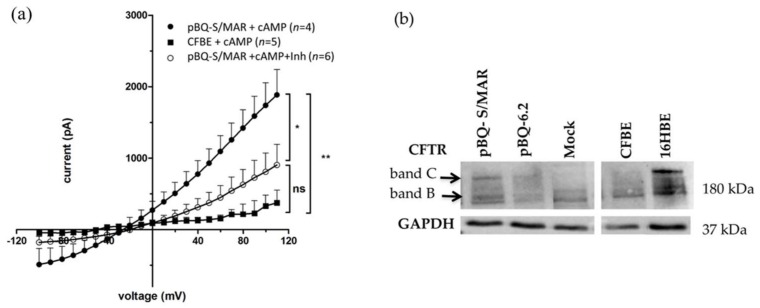
Functional CFTR production in transfected CFBE cells. (**a**) CFTR channel encoded by pBQ-S/MAR increases chloride currents in CFTR deficient cells. The graph shows the average current/voltage relationships in the presence of a cAMP-containing cocktail (+cAMP) of pBQ-S/MAR transfected (pBQ-S/MAR *n* = 4) and un-transfected CFBE cells (CFBE, *n* = 5) or plus 10 μM CFTR_inh_-172 (pBQ-S/MAR + Inh) (*n* = 6). Data are means ± SEM; * *p* < 0.05; ** *p* < 0.01; Student’s *t*-tests. (**b**) Representative western blots of proteins extracted from cells transfected with pBQ-S/MAR or pBQ6.2 grown for 14 days after transfection, and polarized for 9 more days. Controls: mock, CFBE treated with transfection reagent only; CFBE and 16HBE un-transfected cells. Band B, immature form of CFTR; band C, mature CFTR.

It has been previously shown that cells transfected with the parental pBQ6.2 vector generate cyclic adenosine monophosphate (c-AMP)-dependent CFTR chloride channels as assessed by whole-cell patch-clamp [35]. Similarly, the pQB-S/MAR vector, which differs from pBQ6.2 for the addition of the S/MAR-GFP element, generated a CFTR-dependent chloride current in CFBE cells (Figure 4a). Indeed, in the presence of the cAMP activation cocktail, whole-cell patch clamp recordings showed a significant increase in Cl^−^ currents in pBQ-S/MAR transfected cells in comparison to what was observed in un-transfected CFBE cells (pA at +110 mV: 1885 ± 354.6 *n* = 4 vs*.* 374.0 ± 176.4 *n* = 5, respectively). Moreover, Cl^−^ currents recorded from pBQ-S/MAR transfected cells in presence of CFTR_inh_-172, a selective blocker of the CFTR channel, reverted the cAMP-evoked Cl^−^ currents (pA at +110 mV: 903.8 ± 288.9 *n* = 6).

pBQ-S/MAR-driven CFTR expression in CFBE cells was further evaluated by western blotting analysis. For this, CFBE cells transfected with pBQ-S/MAR, pBQ6.2 or without DNA (mock), were grown in non-selective conditions for 7 or 14 days, then seeded on filters and maintained in air-liquid interface (ALI) for additional 9 days to allow cell polarization. 16HBE cells (wt *CFTR*) were used as a positive control. As expected, the mature CFTR band (band C) was detected in the positive control (16HBE) while it was absent in mock-transfected CFBE cells, which showed almost exclusively the immature CFTR band B (Figure 4b). Differently from parental cells, transfected CFBE cells showed the presence of the mature CFTR band C that appeared at higher level in cells containing pBQ-S/MAR compared to those with pBQ6.2.

### 2.4. CFTR Expression in CFBE Cells Improves Tight Junction Organization and Function

**Figure 5:**
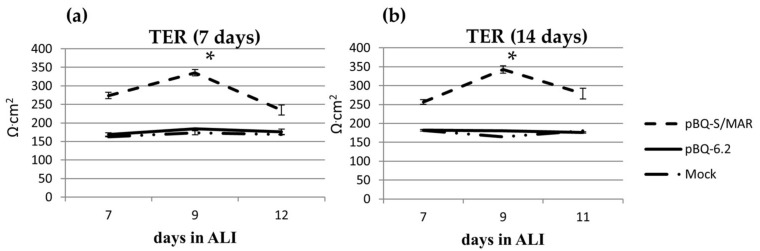
CFTR expression by pBQ-S/MAR increases TER of CFBE cells. TER was measured in CFBE cells, mock or transfected with the indicated plasmids at day 7 (**a**) or 14 (b) after transfection. Data (Ω·cm^2^) are shown as the mean ± SD of three independent experiments. *, *p* <0.05. Statistical analysis unpaired *t*-test pBQ-S/MAR versus pBQ6.2.

It has been previously shown that CFTR, through the multiprotein complex CFTR–NHERF1–ezrin–actin, acts in maintaining tight junction (TJ), organization and barrier function of airway epithelia [11,38]. As trans-epithelial resistance (TER) is mainly due to the shunt conductance, wt CFTR trafficking to the apical membrane and proper TJ organization are expected to reduce paracellular permeability thus increasing TER [38]. Therefore, we considered the epithelial resistance as a surrogate measurement of wt CFTR production and transport to the apical membrane. CFBE cells transfected with pBQ-S/MAR or pBQ6.2 were expanded for 7 or 14 days, then seeded onto filters and shifted to ALI (air-liquid interface) to allow cell polarization. As previously reported, CFBE cells had significantly lower TER than 16HBE (164.64 Ω·cm^2^ ± 4.75 versus 700 ± 25 Ω·cm^2^, *p* = 0.004)). At variance, TER increased in CFBE cells transfected with pBQ-S/MAR (335.44 ± 8.71 Ω·cm^2^) while it remained low in CFBE transfected with pBQ-6.2 (184.24 ± 0.79 Ω·cm^2^). Additionally, no difference was observed between cells expanded for 7 (≈3 cell divisions) or 14 days (≈six cell divisions) after transfection, suggesting that the recovery of TJ organization remained stable in duplicating cells (Figure 5).

**Figure 6:**
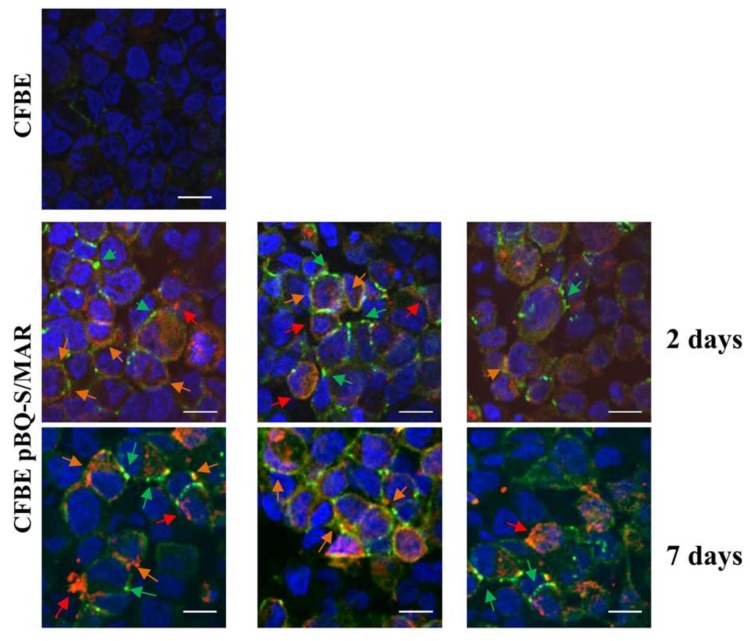
CFTR localizes to the apical membrane in pBQ-S/MAR transfected cells. Cells propagated for 2 or 7 days after transfection were seeded on filters, maintained in ALI conditions for 9 days and probed with anti-CFTR and anti-ZO1 antibodies. At that time the average TER values were 280 Ω·cm^2^ with no significant variations among filters. Secondary antibodies were: green for ZO1, red for CFTR. Nuclei were counterstained with DAPI (blue). Red and green arrows point to CFTR and ZO1 signals, respectively; orange arrows indicate co-localization of red and green spots. Scale bar, 10 μm.

As an increase in TER is likely to reflect the recovery of CFTR trafficking to plasma membrane and TJ organization, we analyzed the localization of CFTR and ZO1 in polarized CFBE/pBQ-S/MAR cells. Results obtained with cells propagated for 2 or 7 days after transfection, showed that ZO1 localized properly to TJ as demonstrated/assessed by typical chicken-wire pattern (Figure 6). Additionally, co-localization of the CFTR and ZO1 signals (orange arrows) could be detected. Interestingly, the localization signals of both CFTR and ZO1, appeared better defined in samples analyzed 7 days after transfection with respect to those detected at day 2. Collectively these analyses strongly suggest that CFTR provided by the pBQ-S/MAR vector ensures long-term correction of CFTR dysfunction and CF-associated defects in bronchial epithelial cells.

## 3. Discussion

Improving the efficacy of gene therapy vectors is still an important goal towards the development of gene therapy treatment for CF patients. The meticulous work done to date, mainly by the UK CF gene therapy consortium, provided plasmid-based vectors with optimized expression cassettes both in the promoters and in the codon usage of the *CFTR* gene [15]. However, a Phase 2b trial with monthly aerosol administrations showed a modest improvement in lung functional tests as compared to the placebo group at 12 months of treatment [17]. More recently, in the attempt to improve the vectors for the lung gene therapy, lentiviruses, showing high transduction efficiency and life-long (2 years) gene expression in the mouse lung, have been developed [22]. However, lentiviral vectors are integrative and potentially genotoxic.

In the context of vector development for CF gene therapy, the present work originates from the idea that S/MAR elements may improve vector persistence and CFTR expression, while reducing possible genotoxic effects due to insertion in the host genome. The S/MAR element, inserted in a transcription unit, through interaction with the nuclear scaffold, promotes replication and episomal maintenance of plasmid- or viral-based vectors, in vitro and in vivo [30,36]. As a preliminary analysis to our work, we studied the maintenance of the S/MAR based pEPI vector in proliferating bronchial epithelial cells, 16HBE and CFBE. In both cell lines, we observed the persistence of extra chromosomal pEPI vector by FISH and by *E. coli* rescue. Additionally, the recovery of similar amount of plasmid DNA from cells grown for 14 or 28 days after transfection suggested that the pEPI developed as an episome in these cells, although we cannot exclude the presence of rare integration events. These results prompted us to assemble the pBQS/MAR vector. The starting material was the first generation CFTR vector pBQ6.2 [37] and the pEPI [29]. The resulting pBQ-S/MAR showed longer persistence as an episome in bronchial epithelial cells as compared to the parental pBQ6.2 as demonstrated by the *E. coli* rescue assay. Indeed, S/MAR episomes were still recovered 14 days after transfection, corresponding to 7 cell duplications. Considering a 1 month turn over for lung epithelial cells, which is exactly the timing of GT treatment for CF [17], this kind of vectors are expected to improve the persistence of the treatments in the lung, which in turn might contribute to reduce the frequency of treatments. However, this important goal is subordinated to the delivery of the vector in progenitor cell populations. This is well documented in Verghese et al. [36], showing that transduction of hematopoietic progenitor cells with and S/MAR hybrid non-integrating lentiviral vector, gives rise to expression of GFP from the episome, in clonogenic colonies and in peripheral leukocytes of transplanted mice. In the future, it is reasonable to hypothesize that combination of S/MAR based vectors and cell therapy with epithelial cell progenitors, would significantly improve the stability of GT treatments in CF patients. However, it has to be considered that episome stabilization is a rare event occurring only in few transfected cells and, similarly to EBV (Epstein–Barr virus)-vectors, appears to be dictated by nuclear localization [39,40]. Importantly, once established pEPI episomes are efficiently maintained as extra-chromosomal DNA circles at 5–10 copies/cell, which contact host chromatin at actively transcribed foci [31]. Interestingly, in some cases, in single-cell derived populations the preferential contact sites greatly exceed the vector copy number suggesting that the episomes may actively change their location in the cell population [31].

The S/MAR vectors have a peculiar architecture comprising a transcription unit with the S/MAR and the EGFP reporter, driven by the cytomegalovirus promoter (pCMV), which has been reported to undergo silencing, at least in hematopoietic cells, possible due to epigenetic modifications [41]. Therefore one question was whether a second transcriptional unit, cloned in the same S/MAR episome, could be active. In our case this was the human CFTR gene whose expression was studied in comparison to the parental, non-S/MAR, pBQ6.2 vector. Functional analyses showed that CFTR expression by the S/MAR-based vector was more stable respect to the parental vector and rescued proper organization of TJs in polarized cells as assessed by epithelial resistance, ZO1 localization and patch clamp. Interestingly, and in accordance with previous study [36], our data suggest that TER analysis, which is a simple, rapid and cost-effective assay, is suitable to test functional CFTR activity in epithelial cells and may help future work for screening CFTR activity. Indeed, by this simple assay we have detected differences in the functional activity of the CFTR provided by the S/MAR or the pBQ vectors.

In summary, the results reported herein suggest that the inclusion of an S/MAR element to a canonical plasmid-based vector improves long term CFTR production and function in polarized epithelial cells opening the possibility that S/MAR elements could contribute to further improve more advanced CF gene therapy vectors such as the pGM169 plasmid or the retroviral rSIV.F/HN-hCEF-CFTR [15]. Additionally, as S/MAR-BACs (bacterial artificial chromosomes) have also been produced, inclusion of the S/MAR element in *CFTR* containing PAC or BAC vectors [42,43] might lead to the development of episomes containing the entire *CFTR* locus, comprehensive of its regulatory elements ensuring natural and cell-specific expression of the therapeutic gene.

Whatever the backbone, based on data reported herein and previous findings, it is reasonable to surmise that S/MAR-CFTR vectors, when inside the cells will be stabilized as episomes, although this will not attain to all but to only a fraction of the transfected cells [39]. Importantly, it was estimated that the correction of as few as 10–20% of the bronchial epithelial cells with wt *CFTR* is sufficient to provide therapeutic levels of CFTR activity [14,15]. Thus, the establishment of CFTR episomes in replicating cells, although occurring in few targeted cells, is expected to promote long-term CFTR expression at therapeutic level. However, detection limits of the methods used to identify extra-chromosomal replicons, such as FISH, do not allow us to exclude the occurrence of rear integration events in cells transfected with S/MAR-based vectors. Properly designed experimental approaches, not yet developed, should be applied to specifically investigate integration events of the S/MAR replicons in clones with established episomes.

Several future modifications are likely to improve S/MAR vectors for the lung gene therapy including the use of specific cis-regulatory elements, substitution of the EGFP reporter with the CFTR gene and the development of selection strategies aimed to augment the fraction of cells retaining the vector. Collectively, this work opens the way to the further development of S/MARS-based CFTR episomes, with the final aim to improve safety and persistence of the treatment in CF lungs.

## 4. Materials and Methods

### 4.1. Plasmids and pBQ S/MAR Construction

Plasmids: pEPI-EGFP (6.7 kb), containing a CMV-EGFP-S/MAR transcription unit was kindly provided by HJ Lipps [29]; pBQ 6.2 (9.16 kb), containing the full length *CFTR* cDNA cloned in pBluescript-SK plasmid [35], was a generous gift of J Rommens. pBQ-S/MAR was assembled in two steps: (1) the pCMV-EGFP-SMAR fragment isolated from the pEPI-EGFP was cloned into the pBQ6.2; (2) the SV40-polyA element was inserted downstream the S/MAR element. A detailed map of the plasmid, named pBQ-S/MAR is reported in Figure S1.

### 4.2. Cells, Transfection and Culture Conditions

Functional studies of the pBQ-S/MAR and its parental vectors, pBQ6.2 and pEPI-EGFP, were done in the bronchial epithelial cell lines 16HBE 14o^-^ (wt *CFTR*) and CFBE41o^-^, homozygous for the CFTR Phe508del mutation), which were kindly provided by Gruenert et al. [44]. Growth medium was MEM medium (Eagle’s Minimal Essential Medium, Euroclone, Pero, Milan, Italy) supplemented with 10% FBS (Fetal Bovine Serum, Euroclone), Penicillin/Streptomycin 0.1 mg/mL and Glutamine 2 mM (Euroclone, Pero, Milan, Italy) in plastics coated with 50% of HAM’S F12medium, 50% of DMEM medium, 10 mg/mL of fibronectin, 29 mg/mL of collagen and 100 mg/mL of BSA (Bovine Serum Albumin, Sigma Aldrich, Saint Louis, MO, USA). Cells were transfected by nucleofection, buffer V, program O-17 (Amaxa Nucleofector II, Lonza, Basel, Switzerland) or Lipofectamin 2000 (Invitrogen, Thermo Fisher Scientific, Waltham, MA, USA) following manufacturer’s specifications. Briefly, 1 × 10^6^ cells/well in 6-well culture plates were seeded the day before transfection and transfected with 3 μg of plasmid DNA for 16–18 h, after which fresh medium was replenished. 48 h after transfection GFP-positive cells in the bulk population (i.e., transfection efficiency) were determined by flow cytometry with a FACS-calibur instrument (BD Bioscience, Franklin Lakes, NJ, USA). Live and dead cells were discriminated by propidium iodide staining.

For air liquid interface (ALI) cultures, cells were seeded onto snapwells (Corning, Corning, NY, USA) at 3 × 10^5^ cells per filter and grown submerged for 2–3 days; when cells become confluent the apical medium was removed and the basal medium was replaced daily. TER was measured using chop stick electrodes and a volt-ohm meter (Millicell^®^-ERS, Millipore, Burlington, MA, USA).

### 4.3. E. coli Rescue

The extracromosomal DNA was isolated from 1 × 10^6^ transfected cells following the protocol “Hirt Supernatant” [45]. Briefly, cells were washed in PBS (phosphate buffer saline) and lysed in 1 mL of lysis solution (10 mM Tris-HCl, 1 mM EDTA, 1% SDS, 200 mg Proteinase K). After 4 h of incubation at 37 °C, 5M NaCl (250 μL) was added drop-wise and the samples incubated at 4 °C for 16–18 h. The aqueous phase was recovered by centrifugation (15,000× *g* for 30 min at 4 °C), extracted with an equal volume of phenol/chloroform/isoamyl alcohol (25:24:1, *v*/*v*) and precipitated with 1 volume of isopropanol; the DNA was recovered by centrifugation, the pellet was washed twice with ethanol 75% and dissolved in sterile ddH_2_O. Electrocompetent *E. coli* cells (strain DH10B) were electroporated with Hirt DNA samples, 1–4 μL, or 1–10 pg of the control plasmid pMAX (Lonza) using the Gene Pulser Electroporator (BIO-RAD, Hercules, CA, USA) as follows: capacitance 25 mF, voltage 2.5 kV, resistance 200 Ω. After recovering in LB medium at 37 °C for 1 h, aliquots were spread on selective agar plates. LB-ampicillin (100 μg/mL) for pBQ-S/MAR and pBQ6.2; LB-kanamycin (50 μg/mL) for the pEPI-EGFP.

### 4.4. RNA and Protein Analysis

RNA was extracted with TRIzol (Invitrogen) following manufacturer’s specifications. cDNA was prepared using the Reverse Transcription system (Promega, Madison, WI, USA) and CFTR and the β-actin were amplified using the following primers: CF7C R, ATAGGAAACACCAAAGATGA; CF17(S) F, GAGGGATTTGGGGAATTATTTG; β-act R, TGGTGACCTGGCCGT; β-act F, GCCGGGACCTGACTGACTA; CF7C R and CF17(S) F, amplified CFTR from 16HBE but not CFBE cells as specific for the wt CFTR transcript (Figure S2). Amplification was carried out under quantitative conditions for 26–28 cycles. Relative quantification of CFTR mRNA was performed as previously reported [46] using the following primers: CFTR-F, AAGCGTCATCAAAGCATGCC; CFTR-R, TTGCTCGTTGACCTCCACTCA; β-actRQ F, GCCGGGACCTGACTGACT; β-actRQ R TGGTGATGACCTGGCCGT.

CFTR protein was detected by western blotting as previously reported [47]. Briefly, cells transfected with plasmid DNA, seeded onto 6-Well Millicell^®^ Hanging Cell Culture Inserts, 0.4 μm pore size, PET (Millipore, Billerica, MA, USA) and grown for up to 9 days in ALI. Cells were lysed in Lysis Buffer (110 mM NaCl, 50 mM Tris-HCl pH 7.4, 0.5% Triton X-100, 0.5% Igepal CA-630, added with protease inhibitors) and 50 μg proteins samples were analysed using the following antibodies: anti-hCFTR monoclonal IgG2A clone 24–1 (R&D Systems, Minneapolis, MN, USA) and GAPDH rabbit polyclonal IgG (FL-335 Santa Cruz Biotechnology, Dallas, TX, USA). Antibody-bound proteins were detected by ECL™ Prime Western blotting detection reagents (Amersham, GE Healthcare Life Science, Little Chalfont, Buckinghamshire, UK) and ChemiDoc (BIO-RAD) imaging.

### 4.5. Immunofluorescence and Confocal Microscopy

pBQ-S/MAR- and untransfected CFBE cells were seeded on filters 48 h after transfection or after 7 days, which account for an average of 3 cell duplications. After seeding, the cells were cultured on filters in ALI conditions up to 9 days. Monolayers were washed three times with PBS, then fixed and permeabilized as previously described [48]. After treatment with blocking solution (2% BSA, 2% FBS in PBS), for 15 min at 37 °C, cells were incubated with CFTR antibody MAB25031 mouse IgG2a (diluted 1:100; R&D Systems, Minneapolis, MN, USA) for 1 h at 37 °C, followed by an incubation with an anti-mouse TRITC (tetramethylrhodamine)-conjugated secondary antibody (diluted 1:100) (Sigma Aldrich, Milan, Italy) for 30 min. After three washes in PBS cells were incubated with FITC (fluorescein isothiocyanate)-conjugated mouse anti-zonula occludens (ZO1) antibody (diluted 1:100; Zymed Laboratories, San Francisco, CA, USA). Nuclei were counterstained with DAPI (4′,6-Diamidine-2′-phenylindole dihydrochloride), rinsed three times with 0.2% BSA and mounted with a drop of Fluorescent mounting medium (Dako, Milan, Italy). Cells were analysed with a Nikon Eclipse Ti-E microscope equipped with a unique Perfect Focus System and coupled to Laser scanning confocal microscope C2 (Nikon Instruments, Florence, Italy). Emission from DAPI, FITC and TRITC was detected with selective filters. Images were acquired and analysed using NIS-elements imaging software (version 4.0, Nikon Instruments, Amsterdam, The Netherland).

### 4.6. Electrophysiology

Whole cell patch-clamp recordings were done using the Axopatch 200B amplifier (Axon Instruments) and the pCLAMP 10.0 and CLAMPFIT 10.0 as acquisition and data analyses softwares, respectively (Axon Instruments, Union City, CA, USA), as previously reported [46]. Briefly, cells seeded at 1 × 10^5^ cells/dish (35 mm dishes) were bathed in the following solution (mM): 140 *N*-methyl d-glucamine; 140 HCl; 2 CaCl_2_; 1 MgCl_2_, 10 HEPES, pH 7.4. cMAP-activating solution (400 μM cAMP, 10 μM forskolin, 1 mM IBMX) was added to the pipette solution containing (mM)140 *N*-methyl d-Glutamine, 40 HCl; 100 l-glutamic acid, 0.2 CaCl_2_, 2 MgCl_2_, 1 EGTA, 10 HEPES, 2 ATP-Mg, pH 7.2. Current was recorded starting from a holding potential of −40 mV; voltage steps were from −110 to +110 mV for 200 ms, with 10 mV increment.

### 4.7. Data Analysis and Statistics

Quantitative data were analyzed using the Prism 4 software (GraphPad Software, San Diego, CA, USA). Data are expressed as means ± standard error of the mean (SEM), and were compared using Student’s *t*-test.

## Supplementary Materials

Supplementary materials can be found at http://www.mdpi.com/1422-0067/19/4/1220/s1.
