# Supplementary material for: Assembly and Functional Analysis of an S/MAR Based Episome with the Cystic Fibrosis Transmembrane Conductance Regulator Gene
Source: Int J Mol Sci. 2018 Apr 17;19(4):1220. doi: 10.3390/ijms19041220 (PMC5979583; doi:10.3390/ijms19041220)
Supplement: Supplementary file 1 [file ijms-19-01220-s001.pdf]

A schematic representation of the assembly of the pBQ-S/MAR vector is reported in figure S1.

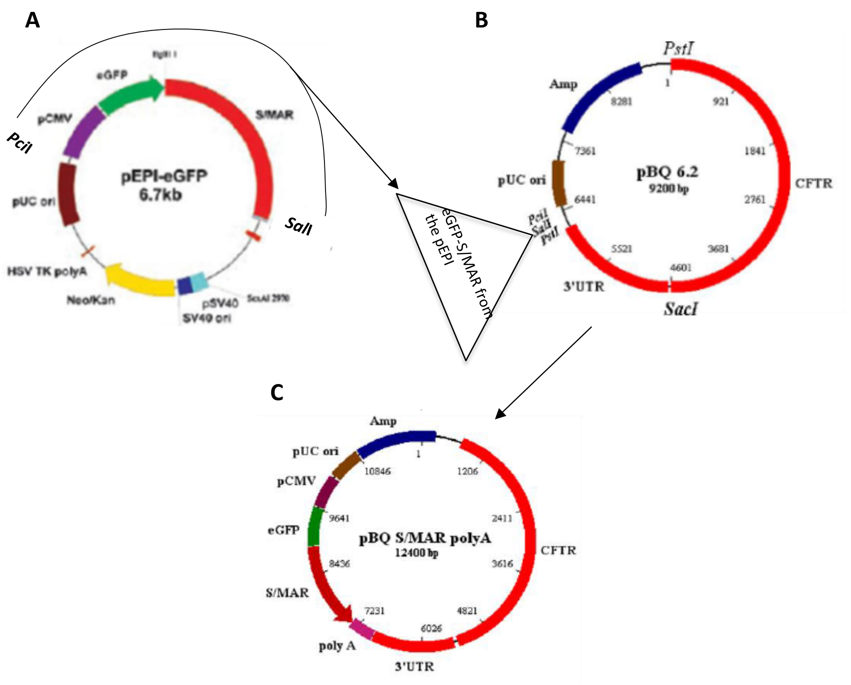

Figure S1. Assembly of the pBQS/MAR vector. **A**) The *Sal*I-*Pci*I fragment, containing the CMV promoter (pCMV), the enhanced GFP gene (eGFP) and the S/MAR element, was cut from the pEPI-eGFP and cloned into the pBQ6.2 (**B**) previously digested with *Sal*I and *Pci*I. Subsequently, a polyA element was inserted as a *Sal*I-*Xho*I fragment downstream the S/MAR element. The resulting pBQS/MAR vector is reported in **C**.

RT-PCR for wt CFTR

Reverse transcription and amplification (RT-PCR) of CFTR with the primer pair CF7C R (ATAGGAAACACCAAAGATGA) and CF17(S) F (GAGGGATTTGGGGAATTATTTG) specifically detected wt CFTR mRNA and not phe508del CFTR transcripts (Illek B. *et al.*, 2008). As expected, RT-PCR of CFBE (homozygous for the phe508del *CFTR* mutation) and 16HBE (wt CFTR) RNA samples clearly showed the presence of the amplification band in latter, but not in the former samples.

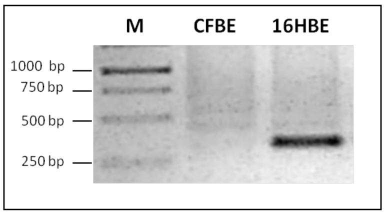

Figure S2. CFTR amplification by RT-PCR with primers CF7C R + CF17(S) F of RNA extracts from CFBE and 16HBE cells.

Illek B, Maurisse R, Wahler L, Kunzelmann K, Fischer H, Gruenert DC.Cl transport in complemented CF bronchial epithelial cells correlates with CFTR mRNA expression levels.Cell Physiol Biochem. 2008;22(1-4):57-68.
